# Colorectal cancer health and care quality indicators in a federated setting using the Personal Health Train

**DOI:** 10.1186/s12911-024-02526-y

**Published:** 2024-05-09

**Authors:** Ananya Choudhury, Esther Janssen, Bart C. Bongers, Nico L. U. van Meeteren, Andre Dekker, Johan van Soest

**Affiliations:** 1 Department of Radiation Oncology (Maastro), GROW Research Institute for Oncology and Reproduction, Maastricht University Medical Centre+, Maastricht, The Netherlands; 2 Department of Orthopaedics, Maastricht University Medical Center+, Maastricht, The Netherlands; 3 Department of Orthopaedic Surgery, VieCuri Medical Center, Venlo, The Netherlands; 4 Department of Nutrition and Movement Sciences, Faculty of Health, Medicine and Life Sciences, School of Nutrition and Translational Research in Metabolism (NUTRIM), Maastricht University, Maastricht, the Netherlands; 5 Department of Epidemiology, Faculty of Health, Medicine and Life Sciences, Care and Public Health Research Institute (CAPHRI), Maastricht University, Maastricht, the Netherlands; 6 Top Sector Life Sciences and Health (Health∼Holland), the Hague, the Netherlands; 7 Department of Anesthesiology, Erasmus Medical Center, Rotterdam, the Netherlands; 8 Topcare, Leiden, the Netherlands; 9 Brightlands Institute for Smart Society (BISS), Faculty of Science and Engineering (FSE), Maastricht University, Heerlen, the Netherlands; 10 Clinical Data Science Group, Faculty of Health Medicine and Life Sciences, Maastricht University Medical Center+, Paul-Henri Spaaklaan 1, Maastricht, 6229 GT Netherlands

**Keywords:** Colorectal, Registry, Quality of Care, Big Data, Privacy

## Abstract

**Objective:**

Hospitals and healthcare providers should assess and compare the quality of care given to patients and based on this improve the care. In the Netherlands, hospitals provide data to national quality registries, which in return provide annual quality indicators. However, this process is time-consuming, resource intensive and risks patient privacy and confidentiality. In this paper, we presented a multicentric ‘Proof of Principle’ study for federated calculation of quality indicators in patients with colorectal cancer. The findings suggest that the proposed approach is highly time-efficient and consume significantly lesser resources.

**Materials and methods:**

Two quality indicators are calculated in an efficient and privacy presevering federated manner, by i) applying the Findable Accessible Interoperable and Reusable (FAIR) data principles and ii) using the Personal Health Train (PHT) infrastructure. Instead of sharing data to a centralized registry, PHT enables analysis by sending algorithms and sharing only insights from the data.

**Results:**

ETL process extracted data from the Electronic Health Record systems of the hospitals, converted them to FAIR data and hosted in RDF endpoints within each hospital. Finally, quality indicators from each center are calculated using PHT and the mean result along with the individual results plotted.

**Discussion and conclusion:**

PHT and FAIR data principles can efficiently calculate quality indicators in a privacy-preserving federated approach and the work can be scaled up both nationally and internationally. Despite this, application of the methodology was largely hampered by ELSI issues. However, the lessons learned from this study can provide other hospitals and researchers to adapt to the process easily and take effective measures in building quality of care infrastructures.

## Background

Methods for acquiring and analyzing real-world big data in the context of health and healthcare are rapidly developing. Real-world and research data in these contexts are often hosted in silos across electronic health record (EHR) systems and are hard to find, share, and interpret by others. One of the key developments to overcome this issue is the Findable Accessible Interoperable and Reusable guiding principles, which are internationally recommended by the G20, European Commission and the European Open Science Cloud. Following these principles would ultimately lead to the internet of FAIR Data and Services, were data, far more divergent than just health and care, can be found, accessed, and (re)used by anyone [1].

Health and healthcare quality indicators are essential measures for assessing the quality of health and healthcare within and between persons and institutions (e.g. hospitals) [2]. National registries, like the Dutch ColoRectal Audit (DCRA) in Netherlands and the Swedish Fracture Register in Sweden collect data from each hospital for the calculation of these quality indicators of patients undergoing colorectal cancer surgery [3, 4]. All Dutch hospitals performing surgery on patients for colorectal cancer submit data to this registry and in return are provided with information on their care quality compared to other Dutch hospitals on a yearly basis. Moreover, these quality indicators can be (re)used to inform patients, insurance companies, and healthcare professionals on the effectiveness of care and consequently act upon this information, in other terms: continuous comparative effectiveness research (CCER) [5].

Current methods for assembling, acquiring, and distributing these data are resource-intensive and do not provide timely information for proper quality management and improvement. Consequently, these methods need radical adaptation using the principles. The current system has several issues that need to be addressed. Firstly, data-entry in quality registries is usually done via “swivel chair integration”, where clinical data (from for instance an EHR) is manually copied to the registry. This requires significant human resources, known as the “registration-assemble burden”. Secondly, quality of the registry data often significantly differs from EHR data, because of erroneous or non-registration of certain data. This leads to over- or underestimating the real quality of care [6]. Thirdly, patient privacy is compromised when data is extracted from the EHR and is shared with third (whether thrusted or not) parties for benchmarking and/or mirroring purposes by national registries [7]. Fourthly, the feedback cycle of registries is too long. Data that is registered and stored today takes up to 1.5 years to be processed and reported back in the form of quality indicators for mirror and benchmark purposes. Because of the long incubation period between data registration and distribution of results, quality registries cannot be used for adequate preference selection and rapid quality improvement, as they may be outdated, incomplete, and faulty.

FAIRification of EHR-data—making them Findable, Accessible, Interoperable, and Reusable—as described by Jacobsen et al. enables efficient use and reduction of risk of errors in EHR data [8, 9]. As such, this tackles the two major issues; 1) the registration-assemble burden and 2) difference in data quality. However, the patient privacy issues, and long feedback time are issues that cannot be resolved only by making data FAIR. Therefore, we propose that when these data are made FAIR they should be linked via a data-infrastructure that enables privacy-preserving federated analysis. Via federated learning, real-world FAIR data can be analyzed locally, meaning they do not leave the silos in which they are stored [10, 11]. The Personal Health Train (PHT) is such an infrastructure, able to visit and analyze local siloes of real-world FAIR data and providing third parties with aggregated results [12–14]. The PHT emphasizes keeping data as close to source as possible, and instead move analytics towards the data [15–17]. This overcomes for the greater part the privacy issue of sharing privacy sensitive data with third parties. Moreover, as analysis ‘trains’ can be sent out at any time, by any trusted party within the infrastructure, near-time calculation of quality indicators and CCER becomes possible. In this study, we will explore the benefits of implementation of FAIR data and PHT for calculation of quality indicators in health and care for patients with colorectal cancer and their caregivers. However, there exists similar infrastructures like the DataShield and Medco2 [18, 19]. While Datashield is restricted to running R based analysis scripts, PHT is enabled to run codes in multiple languages and technology stacks. While PHT employs a central message broker, Medco2 employs a distributed system with no central server.

The aim of this study was to provide a “proof-of-principle” (PoP) concerning FAIRification of EHR-data siloes of patients undergoing surgery for colorectal cancer in two centers and performing federated quality indicator calculations using the PHT infrastructure. Thereby, barriers and facilitators of implementing these relatively new technologies within a real-world hospital environment were described. This facilitates other healthcare centers in applying similar steps and what they need to consider before and during implementation. By doing so, we could move from a local to an (inter)national data-infrastructure and subsequently collectively perform real-time CCER with real-world healthcare data. While the research serves as a proof of concept (PoP), their limitations become apparent when considering a larger scale deployment. For a sustainable deployment across a broader network of hospitals, a more scalable infrastructure with impeccable implementation is necessary.

## Methods

This project aimed to study and provided evidence on the calculation of DCRA-quality indicators from FAIR data endpoints hosted within to Dutch hospitals. We followed a privacy-preserving federated approach for the process, meaning that instead of bringing data to the analyzer, the analysis is pushed to the data. In this section, we first describe the quality indicators and registry data, followed by the steps involved in the process.

### Quality indicators

In this PoP-study, quality indicators 2b and 8 from the DCRA quality registry were calculated [19]. Table 1 lists the quality indicators calculated in this study.

**Table 1:** Quality indicators under consideration

Quality Indicator	Description	Condition	Variables Used
2b	Percentage of patients undergoing resection	Primary Rectum Carcinoma diagnosed within local center (not referred), with waiting time < 5 weeks between diagnosis and therapy	• Hospital ID • Tumor localization • Referred (yes/no) • Date of diagnosis • Date of neoadjuvant therapy • Date of surgery • Type of resection
8	Percentage of patients undergoing a resection	Primary Rectum Carcinoma that had a complication	• Hospital ID • Tumor localization • Referred (yes/no) • Date of surgery • Age • Body Mass Index (BMI) • Charlson Comorbidity Score • American Society of Anesthesiologists (ASA) score • Preoperative tumor complications (yes/no) • Postoperative complications (yes/no)

Quality indicators were calculated using the DCRA quality indicator algorithm, to ensure uniformity of calculation [20]

### Data Extraction Transform and Load (ETL)

The first steps in the process were to collect data from both hospital data repositories, data cleaning, and data hosting in FAIR data repositories. This was important, because data is often scattered across silos within hospitals and may not be interconnected or uniformly organized. Different hospitals use different EHR systems (e.g., SAP (i.s.h.med, Cerner Corp., Den Haag, the Netherlands), HiX (Chipsoft B.V., Amsterdam, the Netherlands), Epic (Epic systems USA, Verona, USA)) and have different data extraction technologies implemented. As such, it is difficult to extract information from these data silos and utilize them for secondary purposes. Hence, the data first needed to be extracted from multiple sources within the hospital, transformed, and—if required—cleaned in order to enhance the value of data and finally loaded into an integrated staging area before making it usable for our study. The two centers involved in this study had different EHR and Business Intelligence systems in place. Table 2 shows the dataset description and the ETL procedures for the two centers.

**Table 2:** Data ETL process

Center	Data Type	Data Extraction Tool	EHR system
Center 1	Structured	Informatica Business Intelligence Tool	Chipsoft (Hix)
Center 2	Structured	SAP Business Warehouse Tool	SAP
	Unstructured / Free Text	Swivel Chair Integration	

### FAIR data model

**Fig. 1:**
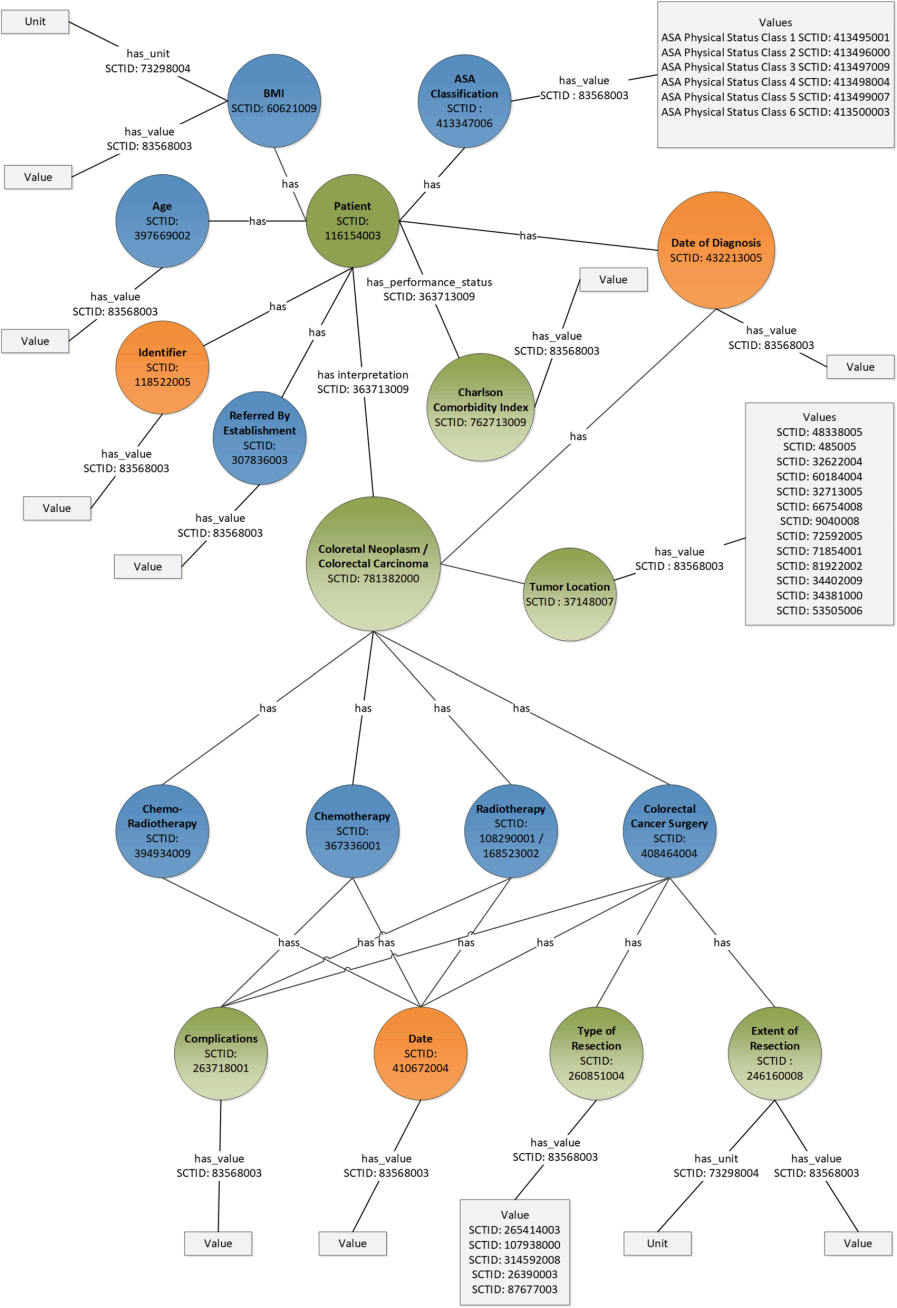
FAIR schema representation of the data variables

**Fig. 2:**
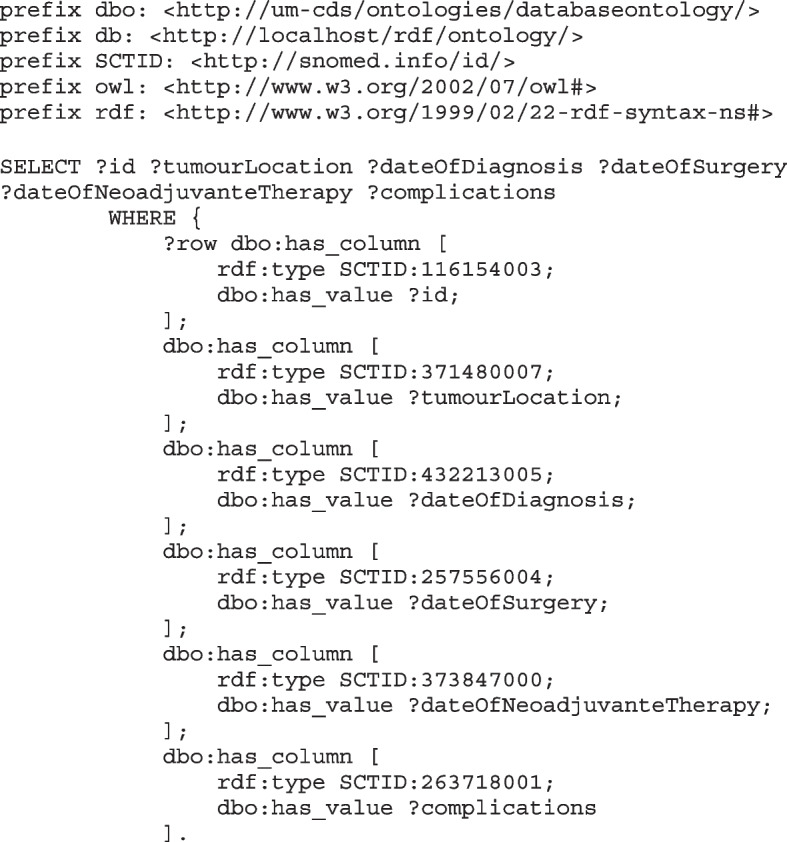
SPARQL Query for retrieving data from the FAIR data stations

Data extracted from hospital EHR systems at each of the two centers were converted into FAIR data and are stored in FAIR data repositories within the hospital. The algorithm in the PHT calculated quality indicators in a data agnostic and privacy-preserving manner (privacy by design provided by the PHT), which means all the calculation and computation within the hospital environment and under the control of hospital IT administration (explained in the next section) [21]Pre. Since, the data stays within the hospital’s environment, the researcher cannot preview the actual data values, but through the FAIR descriptions of the data, acquires knowledge about the metadata and schema only. The query to extract data locally was based on these FAIR data descriptions and is part of the algorithm sent to each data center. The data model followed for converting the data into FAIR data is shown in Fig. 1. The values and variables in the extracted data (in a flat table) were mapped to SNOMED CT terms for maintaining semantic interoperability [22]. The flat table data were then converted to Resource Description Framework (RDF) triples and hosted in a SPARQL endpoint running in a GraphDB instance [23].The flat table converted using TRIPLIFIER, the FAIRification tool and annotated with suitable terminology codes [24]. Each data entity is an RDF triple and can be accessed by a unique and universal resource identifier (uri). The SPARQL endpoint was accessible through the station interface of the PHT only, and as such is hidden from the outside world. The researcher sent a train consisting of the SPARQL query and the algorithm to each center. Figure 2 shows a snippet of the SPARQL query used to retrieve the data from the RDF endpoint [25].

### Federated infrastructure

Calculating quality indicators from institutionally distributed data sets needs a federated infrastructure. The Railway infrastructure (Medical Data Works B.V., Maastricht, The Netherlands), a particular implementation of the PHT, allows the researcher to perform analysis without having to physically collect the data in a central location or server [26]. The components of the infrastructure included a central coordinating server, two data stations holding the FAIR data, the “Train” containing the computation algorithm, and the “tracks” connecting the data stations to the central server. The data station also provided a computation environment for the trains. Each data station authenticates against the server using an authorization token. The server generated an authorization token for each data station and users based on OpenID Connect (OIDC). The researcher needed to authenticate themselves using their personal token and logged in to the system using the username and password given to them by the server administrator. The server provided the researcher with a username and password and an access token, which the infrastructure used to determine if the user was allowed to access the infrastructure. Secondly, the client running at the researcher’s computer received an access token, which authorized the client to post algorithms to the data centers.

The central server was hosted in a cloud virtual machine (2vCPU and 4GB memory, and Microsoft Azure Cloud services). The data stations were hosted in each center and connected to the FAIR data repository (commodity hardware; 2xi5 CPU and 8GB memory. The infrastructure required Docker (Docker inc., Palo Alto, USA) to be installed at each center as a prerequisite and should allow containers to run and execute.

There was a central server connecting the data stations via the internet and accessible via the authentication key received by the researchers from the server administrator. Once these prerequisites were met, the infrastructure was set up and connection testing was completed in approximately 2 days. Since the infrastructure was connected to the FAIR data repository, it was essential to first set up the FAIRification pipeline and load data in the repository. The conversion of data and the query mechanism was explained in the earlier sections.

#### Building trains

**Fig. 3:**
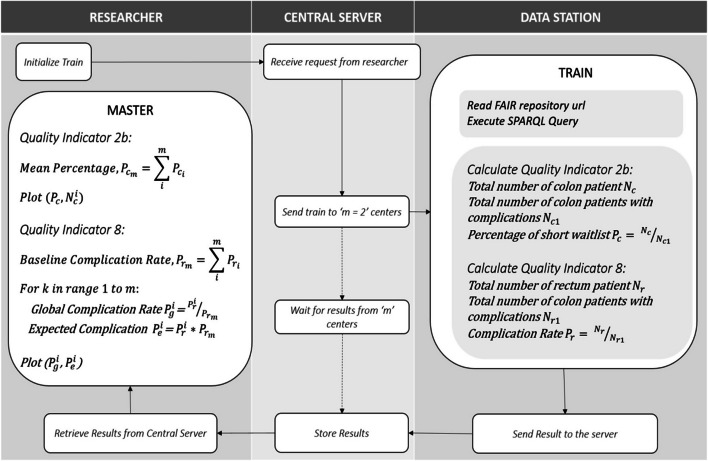
Master and local algorithms (train) in the federated setup

The federated infrastructure can be utilized for all different types of data analysis, like quality assessment, prediction modelling and effectiveness research. The results containing analytics can further be utilized to calculate associations between indicators providing insights for attributional or even casual interpretations in an explorative or even confirmative fashion. The analysis process in this PoP was split in a local analysis and a global analysis algorithm. The local algorithm performed analysis at the data stations, whereas the global algorithm combined these local outcomes to create an aggregated global result. The global result can be either an aggregation of the local results, comparison of two or more local results, or both. The local analysis script was packaged in the form of a Docker container*.* The researcher designed the algorithm for calculating the quality indicators and chose a suitable coding language (i.e., Python) to write the script, although other coding languages could be used as well (e.g., R, MATLAB, STATA). The train consisted of the data retrieval query from the FAIR data stations and the local analysis script. Figure 3 shows the algorithms and the sequence of steps for the federated run.

The SPARQL query to calculate the two quality indicators was based on DICA quality indicators. The quality indicators were visualized in a similar manner to the DICA environment for hospitals.

#### Process evaluation

Firstly, an ‘as is’ and ‘to be’ process mapping was created to show where and how the transition took place. Thereby, time indications of the current and new process were estimated from experience of clinicians within the hospital (current) and results of the current project (new) [27, 28]. During the change process, researchers kept a log of all meetings with stakeholders. From these logs, barriers for implementation of the new process were identified. Recommendations were established describing the strategies applied to overcome these barriers.

## Results

In this section, we present the results obtained from the ETL process and the federated execution of the algorithm.

The ETL process extracted 4699 patients from center 1 and 20 patients from center 2. Because of Ethical Legal Societal Implications (ELSI), real-world data from center 2 could not be extracted. This is further elaborated in the next subsection (“Process evaluation”). Therefore, a simulation dataset of 40 patients, based on actual distribution of the data within center two was constructed. These datasets were stored locally within the respective centers in FAIR data stations. The results obtained from both the centers are presented in Table 3. Table 4 shows the average time required for completing the analysis.

**Table 3:** Results obtained from federated execution of the algorithm

	**Total Population Colon**	**Count of patients with Short Waiting List in Colon Population**	**Percentage of short waitlist**	**Total Population Rectum**	**Count of patients with Complications in Rectum Population**	**Complication Rate**
Center1	546	546	100	4153	2144	0.516
Center 2	10	7	70	7	4	0.571

**Table 4:** Roundtrip time for completion of the federated execution

Time to Task	Unit in seconds
Complete Round trip execution	28
Federated Execution (at 2 nodes)	26
Federated Execution at center 1	26
Federated Execution at center 2	10

**Fig. 4:**
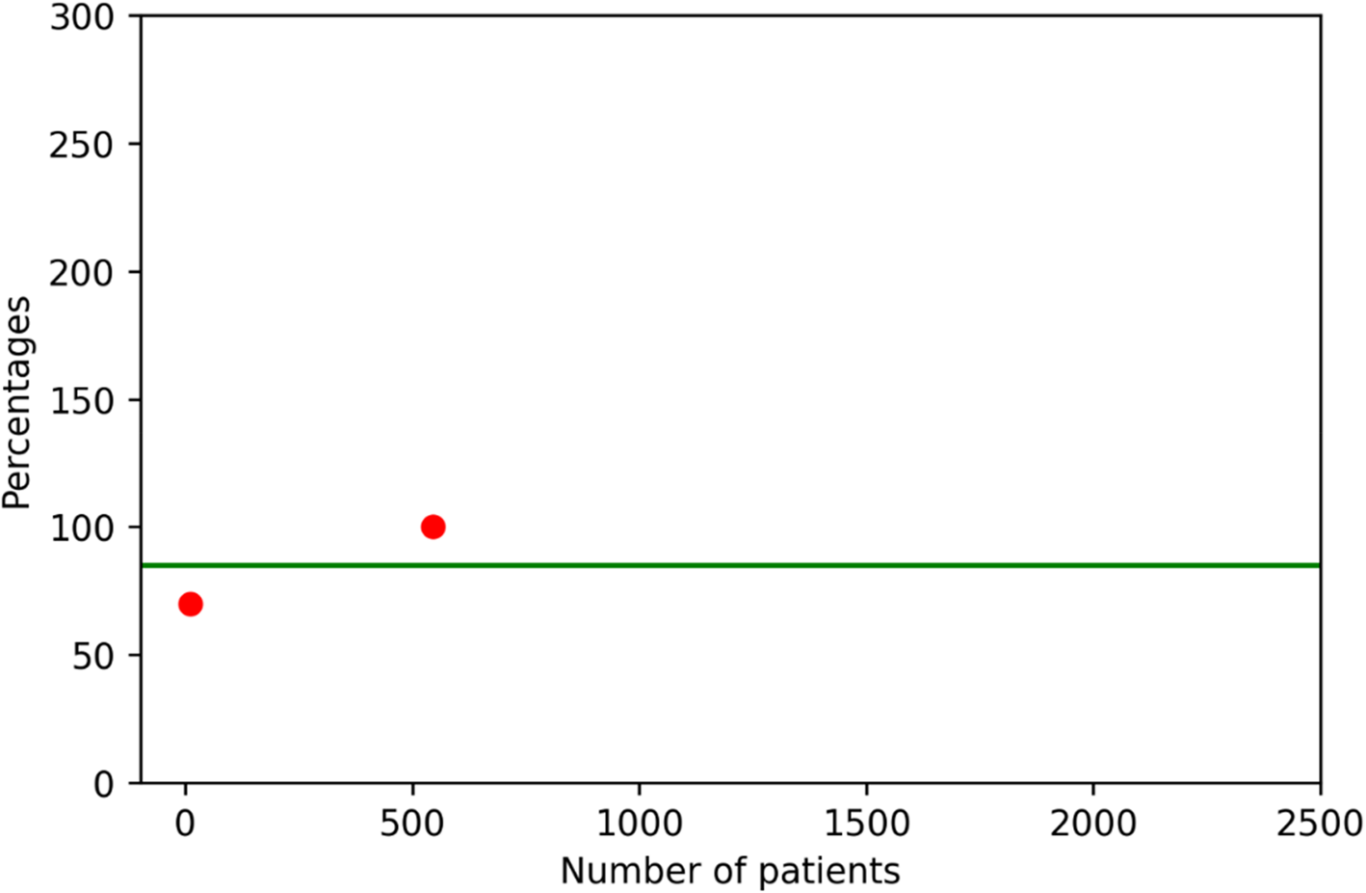
Quality indicator 2b: the percentage of patients undergoing a resection for a primary rectum carcinoma, diagnosed within the local center (not referred), with a waiting period of < 5 weeks between principal appointment and any type of therapy. On the y-axis, the percentage of patients with a time to treatment initiation < 5 weeks is plotted for the individual centers, whereas on the x-axis the number of patients of these centers is plotted. The green line represents the mean percentage of patients with a time to treatment initiation < 5 weeks

**Fig. 5:**
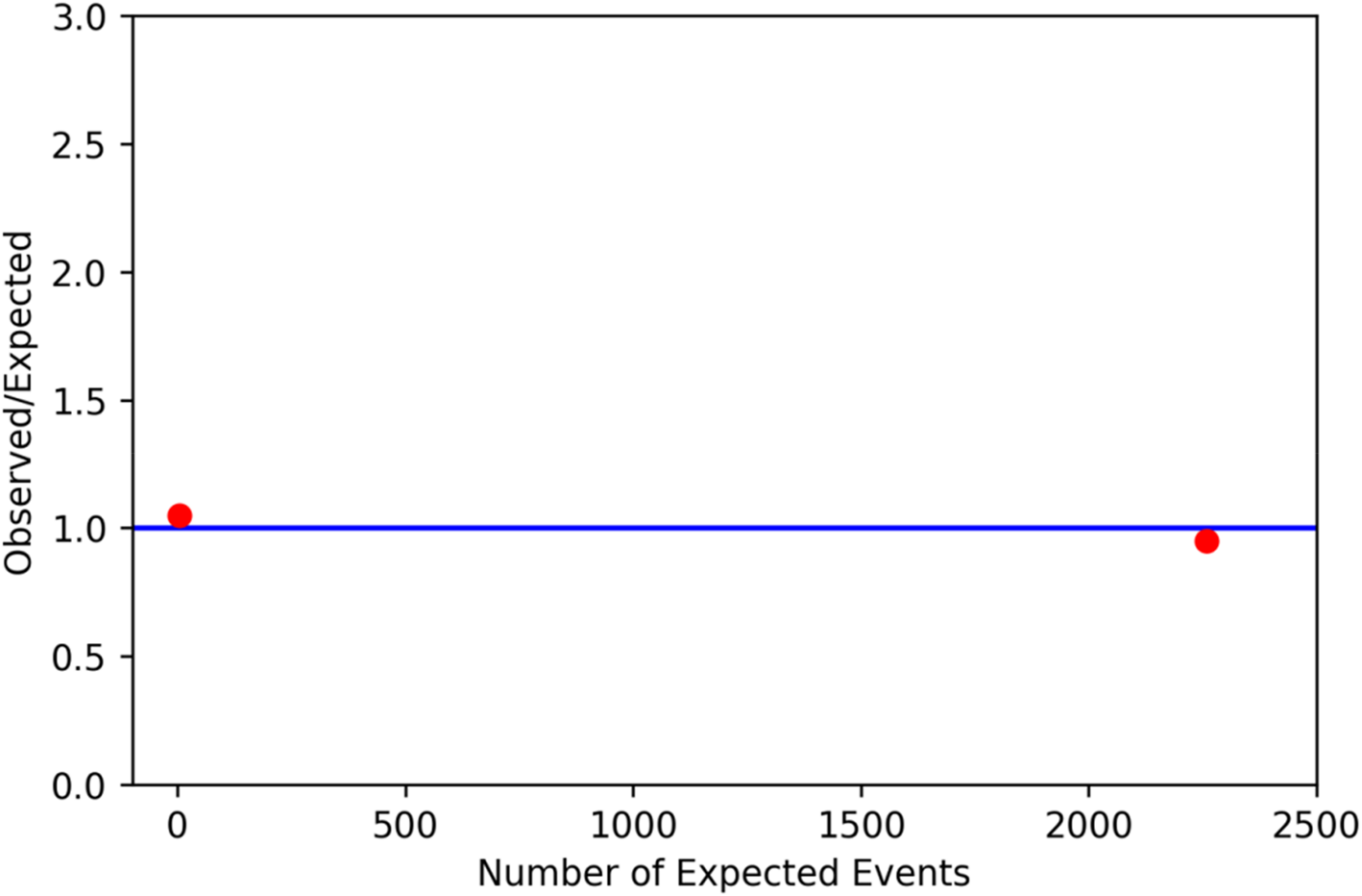
Quality Indicator 8, the percentage of patients undergoing a resection for a primary rectum carcinoma that had a complication. On the y-axis, the percentage of patients with a complication is plotted for the individual centers as a rate of the mean percentage, whereas on the x-axis the number of expected events is calculated by multiplying the mean percentage by the total population of the center. the blue line represents the mean rate of patients with a complication

Finally, the plots for the quality indicators of the two centers calculated in a federated manner by the train are shown in Fig. 4 and Fig. 5. The master algorithm calculated the means of the quality indicators and plotted them together with the individual results into the plots received by the researcher who sent out the algorithm. This is similar to the plots from the DCRA environment that are currently used by clinicians compare their healthcare outcomes to other hospitals in the Netherlands.

### Process evaluation

**Fig. 6:**
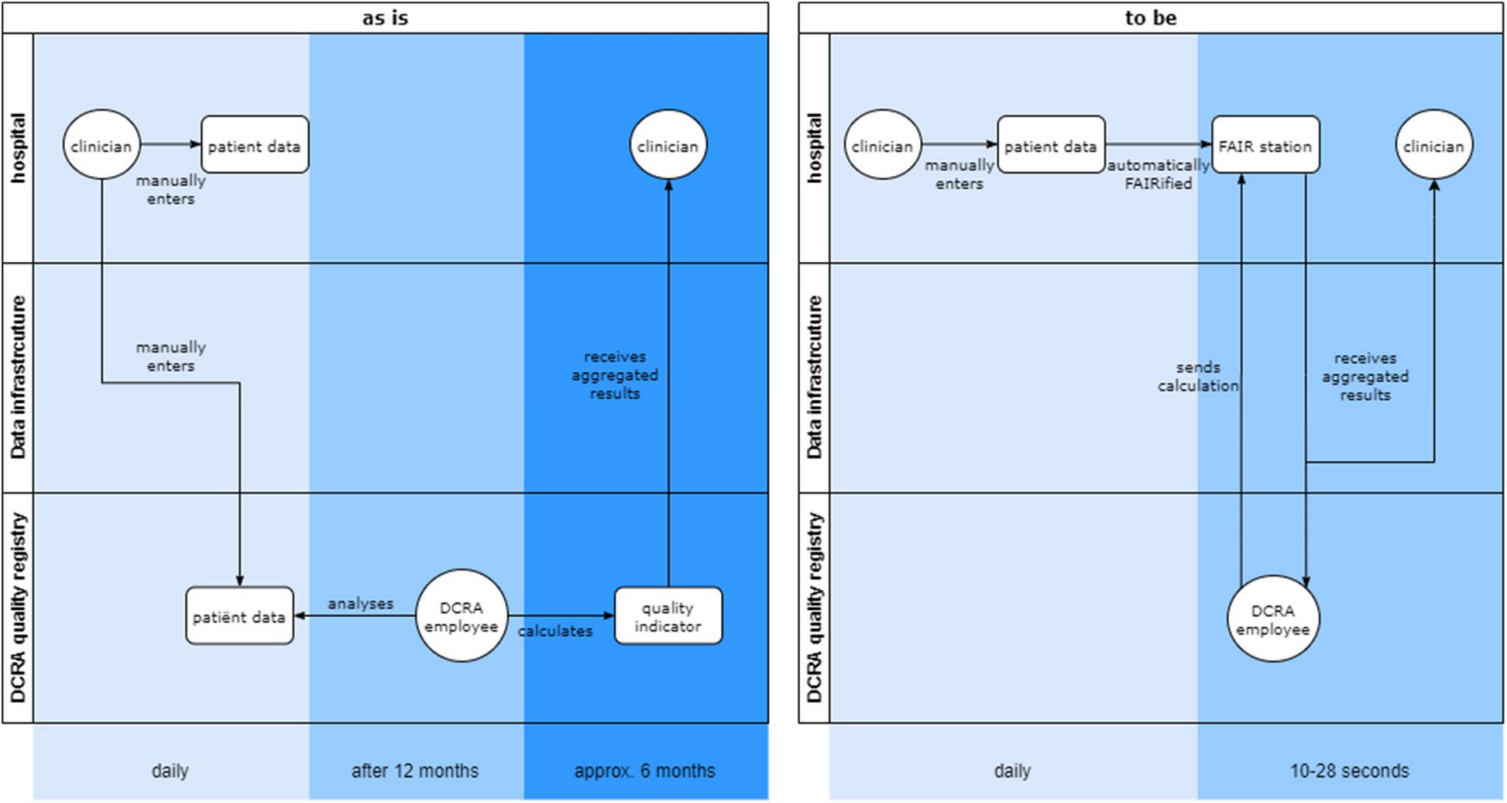
as is’ and ‘to be’ analysis of federated quality indicator analysis

Figure 6 shows the ‘as is’ and ‘to be’ of process of calculating the quality indicators in the old situation and after FAIR and the PHT were implemented, including a timeline.

The process of FAIRification of EHR data and calculating quality indicators using the PHT revealed some important barriers and challenges for application in clinical practice. Both ELSI and practical issues were encountered during the implementation. Major ELSI issues were i) gaining ethical and legal permission to use patient data for secondary but non-research purposes, ii) lack of understanding of FAIR and federated learning within the organization, iii) involvement of many stakeholders who have a say about and interest in the data within the centers, and iv) DICA would not disclose their algorithm for calculating the quality indicators. More practical issues were i) lack of syntactic and semantic interoperability among the data from two centers and ii) deciding whose responsibility it is to standardize and FAIRify data. The hospital administration staff needs to be adequately trained for the FAIRification process. Other practical issues included obtaining a dedicated machine within the hospital where we could set up the infrastructure and the FAIR data endpoint.

To resolve the ELSI issues, review by the local medical ethical committee was performed. Outcome of this review was that it did not fall under the jurisdiction of the local medical ethical committee, as it did not involve the use of data for ‘research’ purposes. In the Netherlands, due to privacy laws, each project involving traceable patient information, a local ‘Data Privacy Impact Assessment’ (DPIA) needs to be completed before the project can be commenced within the center. The DPIA involves a complete assessment of possible risks for privacy violations and measures set in place to prevent this. Finally, this assessment needs to be approved by the local board of directors. Filling out the DPIA is a process involving multiple stakeholders (i.e., privacy officer, lawyer, physician, data expert, and project team). However, due to the lack of familiarity of multiple stakeholders with FAIR and the PHT, this process was very time consuming and involved multiple meetings with stakeholders.

Unfortunately, DICA would not disclose their algorithm for calculation of the quality indicators used in this PoP study. Therefore, we approximated the quality indicators from known information, found in the local DICA environment of the hospitals: which variables were used for calculation and what type of outcome was calculated (e.g., percentage, rate). Thereby, we created a SPARQL query to calculate these outcomes and visualize, like is done by DCRA.

The practical issue of the lack of availability of structured data within center 2 is a more persistent issue that could not be solved within the scope of this PoP. The data used for calculating the quality indicators in center 2 are generally registered in free-text fields within the EHR system. Systems for automatic data extraction, in this case SAP BW, could not be used for extraction of the values. Therefore, swivel chair integration would be the only viable option for short-term data extraction within center 2.

## Conclusion

The aim of this study was to perform a PoP study in which we describe the steps to FAIRification of EHR-systems in real life hospital situations and linking them via the PHT infrastructure to calculate DCRA quality indicators for patients undergoing surgery for colorectal cancer. Thereby, we wanted to provide other centers with means to apply similar strategies within their own center. We found that it is possible to perform FAIRification of EHR data and connect different centers via the PHT infrastructure to calculate quality indicators. This, without individual level data leaving the center. However, we discovered that ELSI issues play a major role in the implementation of such data strategies within a center. Due to ELSI issues and practical issues, it was not (yet) possible to perform the FAIRification step within center 2. Implementation of this strategy to replace the current national quality evaluation strategies can improve accuracy of quality calculation, resolve privacy issues, and facilitate near-time quality improvement strategies upon the calculated results.

During the commencement of this PoP study, multiple ELSI and practical issues were encountered. As usually no process evaluation is done in such PoP studies, it is difficult to foresee and plan for such issues beforehand. By describing these issues, we want to support future endeavors for the implementation of similar strategies in how to approach this in order to make the process less time consuming and burdensome for all stakeholders involved. Main recommendations that can be made are i) early involvement of all stakeholders within the project, ii) setting in place legal measures to protect patient privacy, and iii) taking into account the time and resources needed to perform a DPIA.

As the general concepts of FAIR and the PHT were generally unknown, especially by the privacy officer and lawyers, the commencement of the DPIA was approached very cautiously. This led to a relatively time-consuming process of gaining local approval for conducting this PoP with local data of approximately 6 months. Currently, the full ELSI process has not yet been completed, but will continuously be updated on our gitlab repository [25]. However, we infer that by thoroughly reporting on how to perform such a DPIA and the steps that need to be taken to gain ELSI approval in this PoP will drastically shorten this process in other centers.

In one of the two centers we were unable to automatically extract data directly from the EHR system, and therefore had to resort to the time-consuming swivel chair integration. To improve/facilitate data extraction directly from the EHR, we would recommend i) adoption of semantic standards and ii) coding terminologies at all processes of data management within the hospital. Further adoption of clinical and research standards will further strengthen and streamline such studies [19, 20, 29, 30].

### Obstacles

The project faced many obstacles at different stages mainly from a political, legal, and administrative point of view. As discussed in the previous section, general unfamiliarity of ethical and legal guidelines/stakeholders within the hospitals were a barrier for data extraction and the FAIRification process. Apart from this, the software and tools used in the project had to undergo screening by the IT and legal departments and has proven to be time consuming. Further, the role of infrastructures like PHT have to be explained and often scrutinized from a security point of view. External applications/train computing on local dataset can be seen as a trust issue and proper measurements need to be taken for enhancing trust on the application by the hospital IT.

### Recommendations for follow up

The true value of this work lies in the fact that this project can be scaled up. Firstly, the project can be scaled up to other centers within the Netherlands to create a national infrastructure. This enables a decentralized quality registry providing privacy-preserving near-time calculation of quality of care for patients with colorectal cancer and thereby giving these centers the opportunity of act upon this information in a timely manner. Secondly, the same strategies can be easily applied to multiple quality indicators using more data. This can be done by building on the existing FAIR data model, elaborating the SPARQL query, and using the same PHT infrastructure. Thirdly, this PoP can serve as a blueprint for translation to other populations for which quality indicators are calculated in a national registry, like for patients undergoing other types of surgery or with diseases like for instance Parkinson’s disease.

These scale-ups are facilitated by the outcomes of this PoP and products that were created, like the FAIR data model, SPARQL query, PHT infrastructure, and DPIA files. However, to create a sustainable infrastructure and support for this infrastructure, collaboration with private parties is crucial as healthcare centers mostly do not have the means or want the burden of facilitating such services and tools. Moreover, a preplanned process evaluation of the implementation of such strategies, including expenditures is highly recommended to provide useful information on costs and benefits when implementing these data strategies.

The research will further benefit from considering the energy efficiency of the proposed methodology [31]. This aligns with the growing focus on environmentally sustainable practices in healthcare IT. Further research, informed by studies on energy efficient IoT design could explore methods to optimize the energy footprint of the system [32, 33].

### Conclusion

It is possible to FAIRify local EHR data and to subsequently calculate quality indicators necessary for national quality registries in a privacy-preserving federated manner using the PHT infrastructure. Application of this methodology is largely hampered by ELSI issues. However, lessons learned in this PoP study can provide other hospitals and partners with the means to adapt this process more easily within their center, potentially leading to the creation of a nationwide care quality indicator infrastructure, providing clinicians and hospitals with essential near-time information to correctly and timely monitor and improve their care, with minimal registration burden.

## Data Availability

The software and infrastructure used in the research are available in the below public repositories. 1. Software: Railway https://gitlab.com/medicaldataworks/railway 2. Software: Algorithm (train) https://gitlab.com/UM-CDS/projects/zin-dcra/prototypetrain The study was conducted on sensitive data obtained from Maastricht University Medical Centre + , Maastricht and Maastro Clinic, Maastricht with respective Institutional Review Board approval. In order to obtain access to the data, please contact Dr. Johan van Soest (j.vansoest@maastrichtuniversity.nl).

## Abbreviations

PHT: Personal Health Train
RDF: Resource Description Framework
FAIR: Findable Accessible Interoperable and Reusable
ELSI: Ethical Legal Societal Implications
EHR: Electronic Health Resources
DCRA: Dutch ColoRectal Audit
CCER: Continuous Comparative Effectiveness Research
ETL: Extract Transform Load
DICA: Dutch Institute for Clinical Auditing
DPIA: Data Privacy Impact Assessment
OIDC: OpenID Connect
MATRIX: Matrix Laboratory
STATA: Statistical Software for Data Science
SAP: System, Applications and Products in Data Processing
SPARQL: SPARQL Protocol and RDF Query Language
